# Optical DNA Mapping Combined with Cas9-Targeted Resistance Gene Identification for Rapid Tracking of Resistance Plasmids in a Neonatal Intensive Care Unit Outbreak

**DOI:** 10.1128/mBio.00347-19

**Published:** 2019-07-09

**Authors:** Santosh K. Bikkarolla, Viveka Nordberg, Fredrika Rajer, Vilhelm Müller, Muhammad Humaun Kabir, Sriram KK, Albertas Dvirnas, Tobias Ambjörnsson, Christian G. Giske, Lars Navér, Linus Sandegren, Fredrik Westerlund

**Affiliations:** a Department of Biology and Biological Engineering, Chalmers University of Technology, Gothenburg, Sweden; b Department of Neonatology, Karolinska University Hospital, Stockholm, Sweden; c Department of Clinical Science, Intervention and Technology, Division of Pediatrics, Karolinska Institutet, Stockholm, Sweden; d Department of Medical Biochemistry and Microbiology, Uppsala University, Uppsala, Sweden; e Department of Laboratory Medicine, Karolinska Institutet, Stockholm, Sweden; f Department of Astronomy and Theoretical Physics, Lund University, Lund, Sweden; g Department of Clinical Microbiology, Karolinska University Hospital, Stockholm, Sweden; Medical School, University of Athens; Indiana University Bloomington

**Keywords:** CRISPR/Cas9, optical DNA mapping, antibiotic resistance, intensive care unit, plasmids

## Abstract

The global spread of antibiotic resistance among Enterobacteriaceae is largely due to multidrug resistance plasmids that can transfer between different bacterial strains and species. Horizontal gene transfer of resistance plasmids can complicate hospital outbreaks and cause problems in epidemiological tracing, since tracing is usually based on bacterial clonality. We have developed a method, based on optical DNA mapping combined with Cas9-assisted identification of resistance genes, which is used here to characterize plasmids during an extended-spectrum β-lactamase (ESBL)-producing Enterobacteriaceae outbreak at a Swedish neonatal intensive care unit. The outbreak included 17 neonates initially colonized with ESBL-producing Klebsiella pneumoniae (ESBL-KP), some of which were found to carry additional ESBL-producing Escherichia coli (ESBL-EC) in follow-up samples. We demonstrate that all ESBL-KP isolates contained two plasmids with the *bla*_CTX-M-15_ gene located on the smaller one (~80 kbp). The same ESBL-KP clone was present in follow-up samples for up to 2 years in some patients, and the plasmid carrying the *bla*_CTX-M-15_ gene was stable throughout this time period. However, extensive genetic rearrangements within the second plasmid were observed in the optical DNA maps for several of the ESBL-KP isolates. Optical mapping also demonstrated that even though other bacterial clones and species carrying *bla*_CTX-M_ group 1 genes were found in some neonates, no transfer of resistance plasmids had occurred. The data instead pointed toward unrelated acquisition of ESBL-producing Enterobacteriaceae (EPE). In addition to revealing important information about the specific outbreak, the method presented is a promising tool for surveillance and infection control in clinical settings.

## Abstract (precis)

This study presents how a novel method, based on visualizing single plasmids using sequence-specific fluorescent labeling, could be used to analyze the genetic dynamics of an outbreak of resistant bacteria in a neonatal intensive care unit at a Swedish hospital. Plasmids are a central reason for the rapid global spread of bacterial resistance to antibiotics. In a single experimental procedure, this method replaces many traditional plasmid analysis techniques that together provide limited details and are slow to perform. The method is much faster than long-read whole-genome sequencing and offers direct genetic comparison of patient samples. We could conclude that no transfer of resistance plasmids had occurred between different bacteria during the outbreak and that secondary cases of ESBL-producing Enterobacteriaceae carriage were instead likely due to influx of new strains. We believe that the method offers potential in improving surveillance and infection control of resistant bacteria in hospitals.

## INTRODUCTION

Antimicrobial resistance (AMR) is increasing worldwide and represents a major threat to human health (1). The World Health Organization (WHO) defines AMR as a key priority for reducing mortality due to infectious disease in the Sustainable Developmental Goals from 2014 (2). Hospital settings are particularly vulnerable to the effects of AMR due to the high prevalence of bacterial infections, high antibiotic use, immunocompromised patients, and transmission of bacteria between patients, sometimes with hospital staff as a vector. The increasing prevalence of nosocomial outbreaks caused by extended-spectrum β-lactamase (ESBL)-producing Enterobacteriaceae (EPE) or carbapenemase-producing Enterobacteriaceae (CPE) in high-risk wards, such as intensive care units (ICUs) and neonatal intensive care units (NICUs), is particularly problematic. Around 30% of global neonatal deaths are caused by bacteria resistant to antimicrobials (3), and neonatal sepsis is causing around one million deaths annually (2). Actions to improve surveillance and to rapidly control outbreaks in these settings are essential, since invasive infections with EPE among highly sensitive patients are associated with high morbidity and mortality (4, 5). Methods for identification and genotyping of bacterial isolates have been improved and become faster and more efficient during the last decade. Pulsed-field gel electrophoresis (PFGE) and PCR fingerprinting have been complemented by multilocus sequence typing (MLST) and whole-genome sequencing (WGS). However, currently used methods still require significant hands-on time and usually have the capability of detecting only clonal spread of resistant bacteria. In case of EPE and CPE, horizontal transfer of resistance genes on plasmids presents a further complicating factor, since it will not be detected by traditional typing techniques used at the strain level.

Plasmids are large, circular DNA molecules that are not part of the chromosomal DNA of the bacteria, and in the case of clinical isolates, they often carry multiple resistance genes. For clinical surveillance, traditional methods for plasmid typing are either slow or provide limited information (6). Whole-genome sequencing generates complete genetic information, but short-read sequencing methodologies are unable to generate complete plasmid contigs due to the prevalence of repetitive elements, and in order to obtain complete plasmid sequences for comparison, long-read technologies have to be used, and these methods are still not used in clinical routine as often as short-read technologies. Therefore, there is a need for rapid and straightforward epidemiological tools to detect plasmid transfer in ICU and NICU settings.

We have developed a method, based on optical DNA mapping (7, 8), for characterization of bacterial plasmids. By adding two molecules, the fluorescent YOYO-1 and the non-fluorescent AT-selective netropsin, we form an emission intensity variation along the DNA that reflects the underlying sequence, where AT-rich regions are dark and GC-rich regions are bright (9, 10). To visualize this variation in emission intensity, the DNA barcode, we stretch single intact plasmids in nanochannels and image them using fluorescence microscopy (8). We have demonstrated that the method can with high confidence measure the number and sizes of all large plasmids in a bacterial cell (11) and produce a unique barcode for each plasmid that can be used to identify conjugation of plasmids between different strains and species of bacteria (12–14). The method can also identify previously sequenced plasmids by comparing experimental barcodes with theoretical barcodes of the thousands of plasmids in the NCBI public database (15). In our latest improvement of the assay, we incorporated the CRISPR/Cas9 system to demonstrate on which plasmid a specific (resistance) gene is located (16). Thus, we now have a method that can, in a single experiment, yield information that traditionally requires several different, time-consuming techniques, such as S1/PFGE, PCR, Southern blotting, or long-read whole-genome sequencing. Importantly, our assay can potentially yield sequence information within hours instead of days from initial sample to complete results and is therefore ideal for rapid tracing, for example, in hospital outbreaks.

In this study, we use the assay to study spread of EPE between neonates and potential plasmid spread between colonizing EPE bacteria during a nosocomial outbreak at the NICU at Karolinska University Hospital in Stockholm, Sweden, between November 2008 and March 2009. The outbreak consisted of 17 neonates that were colonized with a low-virulence, highly resistant *bla*_CTX-M-15_-producing Klebsiella pneumoniae (ESBL-KP) strain of sequence type 101 (ST101) (17). Cocolonization was seen with *bla*_CTX-M-15_-producing Escherichia coli in several of the neonates during the 5-year follow-up period after admission from NICU. The goals of the study were to use optical DNA mapping to characterize the resistance plasmids throughout the outbreak and investigate whether the cocolonizing ESBL-producing E. coli were a result of plasmid transfer during the outbreak.

## RESULTS

**FIG 1:**
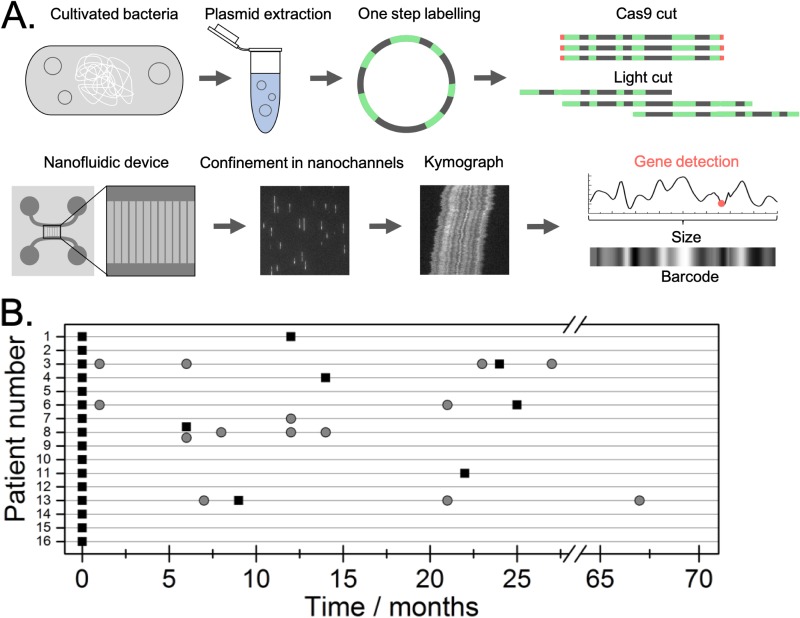
(A) Schematic of the optical DNA mapping protocol used in the study. Plasmids were extracted from cultured bacteria, and the resistance gene was identified using Cas9 targeting *bla*_CTX-M_ group 1. The sample was labeled with YOYO and netropsin in a single step and then introduced into the nanochannels. The plasmids were imaged with a fluorescence microscope, and time traces (kymographs) representing the optical maps were extracted. Kymographs were used to reveal the number of different plasmids in a sample, their size, and on which plasmid the *bla*_CTX-M_ group 1 gene was located (red dot). Optical maps were used to compare plasmids from the different isolates studied. (B) Overview of the different patients and bacterial isolates analyzed. Each patient is listed on the vertical axis, and isolation of EPE is illustrated with black boxes (ESBL-KP) and gray circles (ESBL-EC).

To identify and characterize plasmids carrying ESBL genes (ESBL-plasmids) and investigate possible conjugation events, EPE isolates from the outbreak period were analyzed for plasmid content using the optical DNA mapping protocol summarized in Fig. 1A. 17 neonates were initially diagnosed as carriers of a multidrug-resistant *bla*_CTX-M-15_-producing strain of ESBL-KP sequence type 101 (ST101) (17). However, only 16 strains were viable after storage and could be included in the molecular analysis. For seven of the patients, we also investigated ESBL-KP isolates collected at later stages of the outbreak (6 to 24 months after the first sample was collected), and for four patients, we investigated *bla*_CTX-M_ group 1-positive E. coli isolates collected during the follow-up period (1 to 67 months after the first sample). All isolates, and the time at which they were collected, are illustrated in Fig. 1B. Patient data can be found in an earlier publication (17).

### K. pneumoniae ST101 at the start of the outbreak.

**FIG 2:**
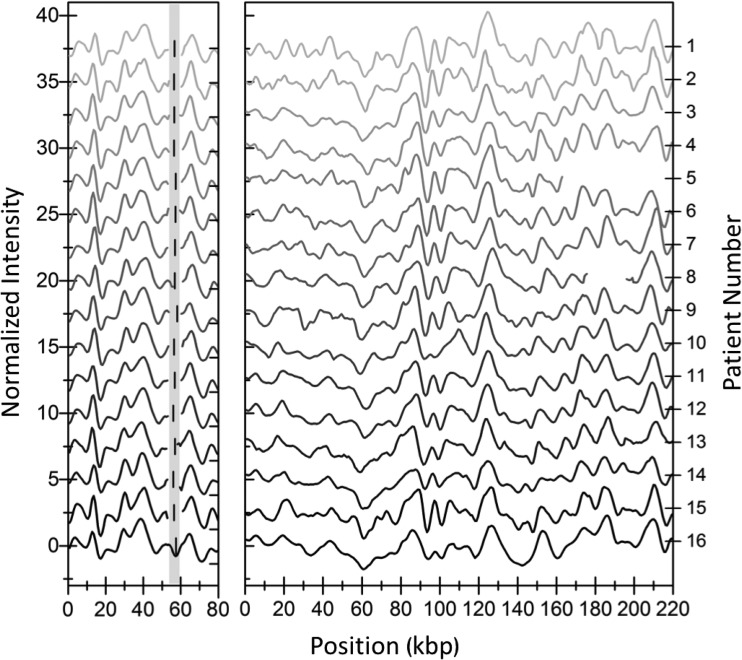
Barcodes of plasmids from the first collected ESBL-KP isolate in each of the 16 patients. (Left) Barcodes of the 80-kbp plasmid. The vertical black line in the shaded region shows where the Cas9 assay predicts the location of the *bla*_CTX-M-15_ gene. (Right) Barcode of the larger plasmid in each isolate. Note that there are deletions in three of the plasmids (patient 3 [5 kbp], patient 5 [55 kbp], and patient 8 [31 kbp]). All barcodes were shifted vertically for clarity.

Figure 2 summarizes the optical mapping results for the 16 ESBL-KP ST101 isolates collected at the start of the outbreak. Each isolate contained two different plasmids, one with a size of approximately 80 kbp (Fig. 2, left) and another with a variation in size between 162 and 222 kbp in isolates from different patients (Fig. 2, right; see also Fig. S1 in the supplemental material). Using the barcodes, we could show that the 80-kbp plasmid from all isolates was cut by Cas9 at the same location in the presence of guide RNA targeting *bla*_CTX-M_ group 1 genes, indicating that this plasmid carried the *bla*_CTX-M-15_ gene (see Table S2 in the supplemental material). By comparing the barcodes of the 80-kbp plasmids from all patients, we could confirm that all these plasmids were identical with a *P* value of ∼0.001 or lower. The *P* values were determined using a set of 1,000 random barcodes, with sizes corresponding to the two compared barcodes (12). The larger plasmid was not cut by Cas9 in any isolate, demonstrating that this plasmid did not carry any *bla*_CTX-M_ group 1 gene. In order to reveal the barcode, we instead linearized this plasmid using light-induced DNA breaks (15). While the larger plasmids varied in size between patient isolates, the barcodes clearly illustrated that they all originated from the same plasmid. A large identical region (∼160 kbp) was present in all isolates, but in three of the isolates, deletions had occurred (patients 3 [∼5 kbp], 5 [∼55 kbp], and 8 [∼31 kbp]). The locations of the deletions are directly visible in the barcodes (Fig. 2, right). This explains the variation in size and exemplifies how optical mapping reveals information that is difficult to assess, or is completely hidden, with other techniques. Taking the deletions into account, we confirmed that all the larger plasmids were of the same origin, with *P* values below 0.001.

10.1128/mBio.00347-19.1 FIG S1 Sizes of plasmids in the ESBL-KP isolates at the start of the outbreak. The light gray bar represents the shorter shared plasmid, and the dark gray bar represents the larger shared plasmid. Download FIG S1, PDF file, 0.2 MB. Copyright © 2019 Bikkarolla et al. 2019 Bikkarolla et al. This content is distributed under the terms of the Creative Commons Attribution 4.0 International license.

10.1128/mBio.00347-19.7 TABLE S2 Statistical confirmation of Cas9 cut position for all ESBL-KP ST101 80-kb plasmids. Download Table S2, PDF file, 0.1 MB. Copyright © 2019 Bikkarolla et al. 2019 Bikkarolla et al. This content is distributed under the terms of the Creative Commons Attribution 4.0 International license.

### K. pneumoniae ST101 in follow-up samples.

**FIG 3:**
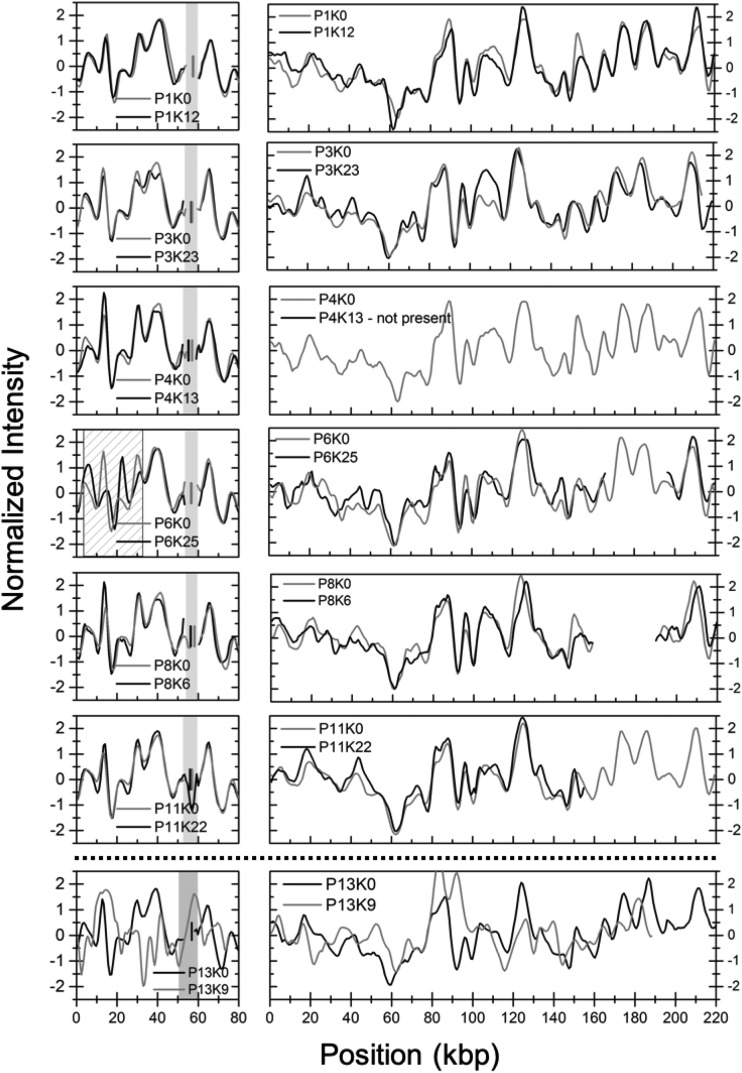
Barcodes from follow-up ESBL-KP isolates (black lines) compared to the corresponding first (gray lines) ESBL-KP ST101 isolate collected. The vertical gray (initial) and black (later) lines in the shaded region show where the Cas9 assay predicts the location of the *bla*_CTX-M-15_ gene. The dashed region indicates the suggested inversion in sample P6K25. For the larger plasmid regions, 5 to 65 kbp in size are missing in four of six isolates, and in one isolate, the plasmid is missing completely. P13K9 contained three plasmids, two of which are shown in the figure and none of the three are the same as the plasmids in the initial P13K0 isolate. Furthermore, we did not identify a *bla*_CTX-M-15_ gene in any of these three plasmids.

Next, we investigated ESBL-KP isolates from seven of the patients (patients 1, 3, 4, 6, 8, 11, and 13) collected 6 to 24 months after the initial isolate (see Fig. S2 for plasmid sizes). The 80-kbp plasmid, carrying the *bla*_CTX-M-15_ gene, was present in six of the isolates investigated. For five of the patients (patients 1, 3, 4, 8, and 11), the 80-kbp plasmids were identical to those at the first time point with a *P* value below 0.001. However, for patient 6, visual inspection of the barcodes suggests that an inversion of about 31 kbp had occurred (dashed region in Fig. 3). The *P* value for this pair of plasmids was significantly higher than for any other combination of 80-kbp plasmids (∼0.003). The larger plasmid was found in five of six isolates (absent in the isolate from patient 4) and was again more dynamic with two isolates containing deletions of 33 kbp (patient 6 [P6]) and 65 kbp (patient 11 [P11]) in size, respectively. For patient 3, the second isolate contained a full 215-kbp plasmid compared to the initial isolate that had a 5-kbp deletion. The barcodes suggest that the deletions have occurred in the same general region in all plasmids (Fig. 2 and 3). All *P* values for these comparisons were again below 0.001. For patient 11, a sample collected at 26 months showed larger structural variations in the larger plasmid, as can be seen in Fig. S3.

10.1128/mBio.00347-19.2 FIG S2 Sizes of plasmids in the ESBL-KP isolates in follow-up samples. The light gray bar represents the shorter shared plasmid, and the dark gray bar represents the larger shared plasmid. The dashed black bars represent the isolate that are unrelated to the other plasmids. Download FIG S2, PDF file, 0.1 MB. Copyright © 2019 Bikkarolla et al. 2019 Bikkarolla et al. This content is distributed under the terms of the Creative Commons Attribution 4.0 International license.

10.1128/mBio.00347-19.3 FIG S3 Structural variations in the large plasmid collected at 26 months in patient 11 compared to the same plasmid in the initial isolate. Download FIG S3, PDF file, 0.1 MB. Copyright © 2019 Bikkarolla et al. 2019 Bikkarolla et al. This content is distributed under the terms of the Creative Commons Attribution 4.0 International license.

The isolate from patient 13 (P13K9) collected 9 months after the initial isolate contained three plasmids with sizes of 72 ± 3.6, 122 ± 6.3, and 204 ± 10.2 kbp. In Fig. 3 (bottom), two of the plasmids are compared with the plasmids in the initial isolate in patient 13. There is no overlap between these plasmids and also not with the third plasmid of intermediate size. Furthermore, we did not find a *bla*_CTX-M_ group 1 gene on any of these plasmids. This isolate therefore represents a second ESBL-KP that was unrelated to the outbreak strain.

### Genetic characterization of the ESBL-KP ST101 isolates.

**FIG 4:**
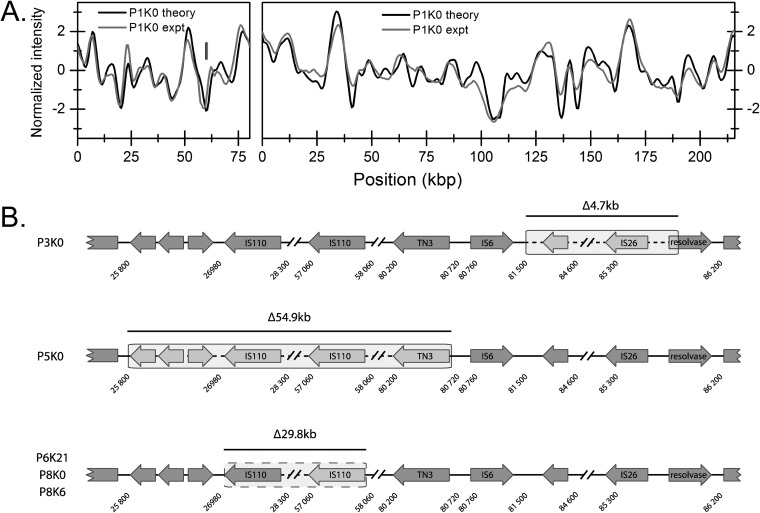
(A) Comparison of experimental optical map (gray) and theoretical optical map created from PacBio sequencing (black) for the two plasmids in P1K0. The vertical lines mark the locations of the *bla*_CTX-M-15_ gene experimentally determined (gray) and predicted from the theoretical sequence (black). (B) Illustration of the positions of deletions in several ESBL-KP isolates. The shaded boxed areas indicate the extension of the deletions.

The optical mapping method provided an overview of the plasmid content in all ESBL-KP samples investigated. To characterize the genetics of the plasmids in the outbreak clone further, we used long-read sequencing on the PacBio platform to sequence the plasmid from the initial ESBL-KP isolate from patient 1 (P1K0). Figure 4 compares the barcodes obtained using optical mapping with the barcodes predicted from the PacBio sequencing, demonstrating excellent overlap. The size of the smaller plasmid was 80,272 bp, and the larger plasmid was 215,872 bp. The position of the *bla*_CTX-M-15_ gene on the smaller plasmid was perfectly predicted from the Cas9 restriction. From the sequence, we determined that the 80-kbp plasmid contained an IncR and an IncFIA origin and, in addition to the *bla*_CTX-M-15_ gene, also contained genes encoding resistance to β-lactams (*bla*_TEM-1_), fluoroquinolones (*qnrB1*), sulfonamides (*sul2*), trimethoprim (*dfrA14*) and aminoglycosides [*aac(*3*)-IId*, *aph(*6*)-Id*, *aph(3*′′*)-Ib*]. The 215-kbp plasmid belonged to the IncFII group and did not contain any resistance genes but two operons for heavy metal resistance (arsenic and copper) and several putative virulence genes such as an urea transport system previously linked to survival in the urinary tract (18). In addition to the two large plasmids, two small cryptic plasmids of 4,572 bp and 4,163 bp were present. These plasmids did not contain any resistance or virulence genes. The characteristics of the plasmids are listed in Table S3.

10.1128/mBio.00347-19.8 TABLE S3 Descriptions of plasmid sequences from whole-genome sequencing. Download Table S3, PDF file, 0.1 MB. Copyright © 2019 Bikkarolla et al. 2019 Bikkarolla et al. This content is distributed under the terms of the Creative Commons Attribution 4.0 International license.

From the complete plasmid sequence, we could also investigate the genetic rearrangements found in the 215-kbp plasmid in several of the ESBL-KP ST101 isolates. Through targeted PCR followed by Sanger sequencing, we could determine the exact deletion breakpoints. As depicted in Fig. 4B, the endpoints of all deletions were associated with insertion sequences or transposons. In three isolates (P6K25, P8K0, and P8K6), a 29.8-kbp deletion was due to homologous recombination between two directly repeated IS*110* elements. Isolates P3K0 and P5K0 had deletions of 4.7 kbp and 54.9 kbp, respectively, both without obvious homologous regions but with endpoints close upstream of a Tn*3* and an IS*26* element, respectively. Most likely, these deletions were an effect of transposase activity. The sequencing results confirmed the optical mapping data regarding both position and size of the deletions. For the deletion in isolate P11K22 and the inversion in the 80 kbp plasmid in isolate P6K25, PCR could not identify the exact endpoints of the rearrangements.

### Investigating potential plasmid conjugation between species.

**FIG 5:**
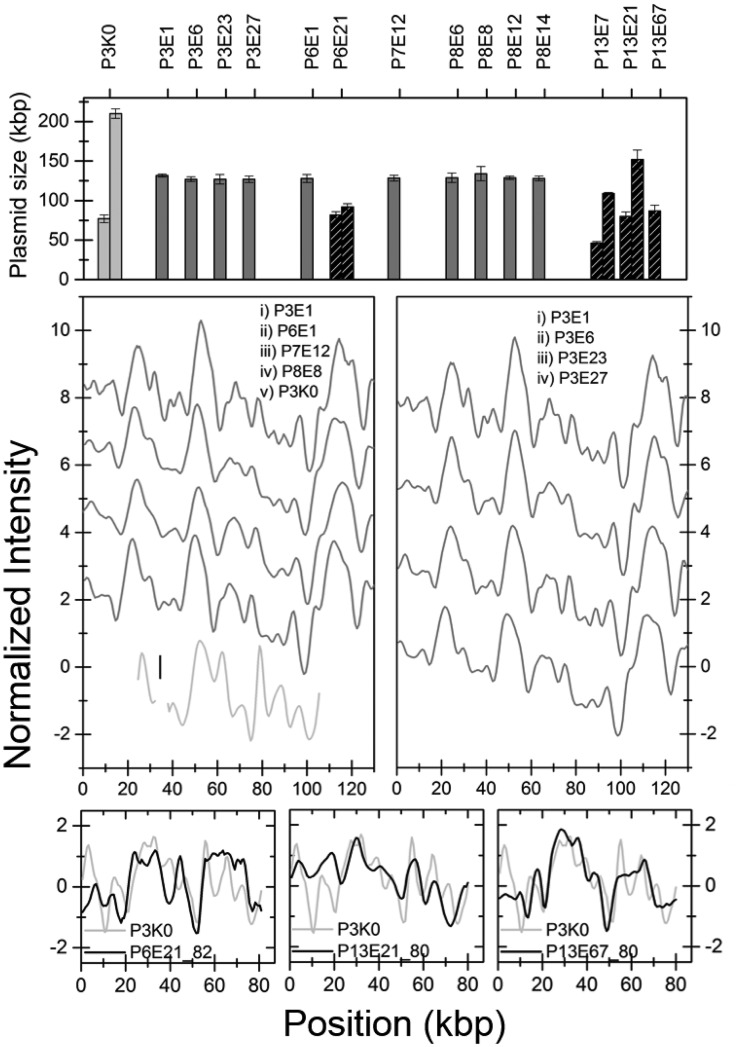
(A) Histogram of sizes of plasmids in all E. coli isolates investigated. The dark gray bars represent isolates carrying the same plasmid, studied in detail in panels B and C. The black hatched bars are isolates with unique plasmid content, and some of these are shown in panels D to F. The light gray columns are from the reference ESBL-KP isolate P3K0. (B) Barcodes of the 129-kbp plasmid in the E. coli isolates with a common plasmid size from different patients (dark gray). The plasmid carrying the *bla*_CTX-M-15_ gene in P3K0 is also shown for comparison (light gray). (C) The 129-kbp plasmid in patient 3 at different time points. The lines are shifted vertically for clarity. (D to F) Comparisons between the 80-kbp plasmid carrying the *bla*_CTX-M-15_ gene in P3K0 (light gray) and plasmids of similar size in the ESBL-EC isolates (black).

In several of the patients, ESBL-producing E. coli (ESBL-EC) bacteria were isolated at different time points during the follow-up period (Fig. 1B). We analyzed the plasmid content by optically mapping 14 ESBL-EC isolates from the five patients where the ESBL-EC at least at one time point dominated the intestinal flora. Figure 5A shows a histogram with the sizes of the plasmids found in all ESBL-EC isolates. No *bla*_CTX-M_-group 1 gene was found on any of these plasmids, giving a direct indication that the ESBL phenotype of these isolates had a different genetic origin than the ESBL-KP. Furthermore, there was no similarity between these plasmids and the plasmids in the ESBL-KP isolates, indicating that the ESBL-ECs had separate origins.

Ten of the investigated ESBL-EC isolates from patients 3, 6, 7, and 8 had identical plasmids (*P* value < 0.001; data not shown) with a size of approximately 130 kbp (Fig. 5B and C; data not shown). PFGE analysis verified that these patients were colonized by the same ESBL-producing E. coli strain (17). To support the optical DNA mapping data, we performed PacBio sequencing on one of these isolates (P3E6). This resulted in one 129,195-bp plasmid of IncFII and IncFIB origin and a small cryptic plasmid of 5,878 bp. None of these plasmids contained any resistance genes, but the large plasmid carried putative virulence genes mainly encoding pili and adhesion proteins (Table S3). The barcode of the 129-kbp plasmid displayed an excellent overlap with the barcode predicted from sequencing (Fig. S4). The results also demonstrate that the *bla*_CTX-M-15_ gene was located on the E. coli chromosome, in agreement with the lack of Cas9 cleavage of any plasmids.

10.1128/mBio.00347-19.4 FIG S4 Comparison between experimental optical map (gray) and theoretical optical map obtained from PacBio sequencing (black) the 130-kbp plasmid in the E. coli isolate in patient 3. Download FIG S4, PDF file, 0.1 MB. Copyright © 2019 Bikkarolla et al. 2019 Bikkarolla et al. This content is distributed under the terms of the Creative Commons Attribution 4.0 International license.

Plasmids from one ESBL-EC isolate from patient 6 and three isolates from patient 13 did not match any plasmids from the other ESBL-EC isolates. Cas9 analysis confirmed that none of these isolates contained any plasmids carrying the *bla*_CTX-M_ group 1 gene and none of the plasmids shared any sequence identity between these isolates or the *bla*_CTX-M-15_ plasmid in the ESBL-KP isolates. To illustrate this, the barcodes of these plasmids were compared with experimental optical maps for the 80-kbp plasmid from the initial ST101 strain (examples in Fig. 5D to F). When comparing the plasmids in Fig. 5D to F, they were stretched to the same length, i.e., the average of the two determined lengths. The only similarity observed between samples was that the plasmid characterized in Fig. 5B and C showed a significant match (*P* value < 0.01) with the 152-kbp plasmid in P13E21 (Fig. S5). Since these plasmids did not carry the *bla*_CTX-M_ group 1 gene, this was not analyzed further.

10.1128/mBio.00347-19.5 FIG S5 Similarity plot for 130-kbp plasmid in P3E0 and 152-kbp plasmid in P13E21. Download FIG S5, PDF file, 0.1 MB. Copyright © 2019 Bikkarolla et al. 2019 Bikkarolla et al. This content is distributed under the terms of the Creative Commons Attribution 4.0 International license.

## DISCUSSION

The goal of this study was to illustrate the use of optical DNA mapping, including Cas9-assisted gene identification, for rapid and detailed characterization of plasmids from bacterial isolates during a resistance outbreak at two NICUs at Karolinska University Hospital in Stockholm, Sweden. The purpose was to characterize the ESBL-plasmids of the outbreak and reveal whether the presence of several different ESBL-producing bacterial species was due to horizontal plasmid spread.

With the introduction of the Cas9 protocol, we were able to directly identify on which plasmid the *bla*_CTX-M_ group 1 gene was located and through the optical DNA barcode we could determine whether this particular plasmid was present in any other isolates during the outbreak. The *bla*_CTX-M_ group 1 producing plasmid was identical in all isolates at the start of the outbreak and was also present in children years after discharge from the NICU. Using optical DNA mapping, we could also show that all ESBL-EC and one ESBL-KP carried different plasmids than the outbreak ESBL-KP, illustrating that these originated from introduction of other ESBL-encoding strains and not horizontal transfer of the ESBL-plasmid. We demonstrated that four patients carried ESBL-EC isolates with the same plasmid, suggesting a small second chain of transmission, but where the *bla*_CTX-M-15_ gene was located on the chromosome. We can therefore conclude that this outbreak was not further complicated by additional plasmid-mediated spread of resistance. Early identification of transmission of resistant strains is highly important in any environment, but particularly important in the vulnerable population of NICU patients. Importantly, while conjugation of plasmids did not occur in this outbreak, we have demonstrated the potential of the method to detect such events in previous studies (12–14).

Complementing our analysis with long-read PacBio sequencing of key isolates demonstrated that the overlap between experimental and theoretical barcodes (15, 19) was excellent. This comparison confirmed that the location of the *bla*_CTX-M-15_ gene along the barcode of the smaller plasmid, predicted by Cas9 restriction, was correct. Furthermore, we could reveal information about the plasmids in all ESBL-KP isolates by performing long-read sequencing on only one key isolate. Long-read sequencing demonstrated that this plasmid carried several resistance genes in addition to the *bla*_CTX-M-15_. For the larger of the plasmids in the ESBL-KP strain, optical mapping revealed that large deletions had occurred in several isolates, both at the start of the outbreak and in follow-up samples, and the positions of these deletions could be verified from the sequence.

In conclusion, we present a novel method, based on optical DNA mapping, for detailed characterization of plasmids and their dissemination during a nosocomial resistance outbreak. The method reveals the number and size of plasmids in bacterial isolates, a barcode for tracing the plasmids, and the presence and location of any resistance gene of interest by straightforward design of guide RNA for Cas9 cleavage. We identified the plasmid carrying the *bla*_CTX-M-15_ resistance gene in all isolates and concluded that no plasmid conjugation had occurred during the outbreak. The combination of optical DNA mapping and long-read sequencing enabled analysis of a large set of samples by sequencing only key isolates. A novel molecular tool, such as the one presented herein, could be highly useful for curbing the spread of antimicrobial resistance in hospitals. Such measures are needed to reach WHÓs Sustainable Development Goals for reducing neonatal mortality (2).

## MATERIALS AND METHODS

### Bacterial samples and molecular analyses.

Intestinal EPE were sampled from 17 patients at the start of the outbreak. Fecal samples were thereafter collected from 14 of the surviving neonates at their discharge from the NICU and every 2 months until 2 years of age. A 5-year follow-up of EPE colonization was, due to drop-outs, conducted on 10 children. Rectal agar gel swabs were taken (Copan, Brescia, Italy) and plated on ChromID ESBL agar (bioMérieux, Craponne, France). Species identification was performed with Vitek2. The *bla*_CTX-M_ group was identified with a previously described (20), probe-based PCR assay. The clinical details of the outbreak are published elsewhere (17). The study was approved by the regional ethical review board in Stockholm, Sweden (2009/734-31/4 and 2014/491-31/3).

### Optical DNA mapping of plasmids.

Bacteria were grown in 100-ml overnight cultures in LB broth, and plasmids were prepared from the cultures using the Macherey-Nagel NucleoBond Xtra Midi preparation kit according to the manufacturer’s instructions. Eluted plasmid DNA was precipitated with isopropanol, and resuspended in 1× TE buffer. Plasmids were treated with Cas9 and guide RNA (gRNA) targeting *bla*_CTX-M_ group 1 by following our previously reported protocol (16). Plasmids and λ DNA (New England Biolabs) at equal concentrations were stained with YOYO-1 (Invitrogen) at a molar ratio of 1:2 (YOYO-1:bp) and with netropsin (Sigma-Aldrich) at a molar ratio of 100:1 (netropsin:YOYO-1) to obtain the DNA barcode. λ DNA was used as an internal size reference (48,500 bp). DNA (2 μM) was incubated with YOYO-1 and netropsin in 10 μl of 0.5× TBE at 50°C for 30 to 60 min in order to achieve uniform staining (21). Samples were diluted with Millipore water to obtain a final concentration of 0.05× TBE and 0.2 μM (bp) of DNA. To suppress photo nicking and photo bleaching of DNA molecules, 2.5% (vol/vol) of β-mercaptoethanol (BME) was added to the solution.

Nanofluidic channels were fabricated in oxidized silicon, as described elsewhere (22). Nanochannels of dimensions 100 nm by 150 nm^2^ and a length of 500 μm were used to stretch the DNA molecules. Prior to loading the samples, the channels were cleaned with a 5% (wt/wt) sodium hypochlorite solution and then washed a few times with loading buffer (0.05× TBE). By cleaning with sodium hypochlorite solution and washing with buffer, some nanofluidic chips were used more than 50 times, and on average, each section of the chip was used at least 10 times. Fifteen microliters of DNA solution was placed in one of the reservoirs, and DNA molecules were forced into the nanochannels by applying pressure with nitrogen gas. An inverted microscope (Zeiss AxioObserver.Z1) equipped with either a 63× (with 1.6 times optovar) or 100× oil immersion objective (Zeiss) (numerical aperture [NA] of 1.46) and an electron-multiplying charge-coupled-device (EMCCD) camera (Photometrics Evolve or Andor iXon) was used to image the DNA molecules. Both circular and linear molecules were stretched in the nanochannels and imaged for 200 frames with 100-ms exposure time.

The data were analyzed in order to detect the number of plasmids in each sample, their corresponding size, as well as their sequence-specific barcode and potential presence of a resistance gene (*bla*_CTX-M_ group 1). In short, the internal size reference λ DNA was used in order to estimate the plasmid size, either directly from linearized plasmids, or from plasmids in their circular form, using a conversion factor of 1.8 (23). Linear fragments of similar size (±10%), obtained either by Cas9 linearization, or via extensive light exposure (15), were compared, and individual barcodes with a high degree of similarity (typically Pearson cross correlation [CC] of >0.75) were merged into a consensus barcode. The consensus barcode was used both for comparison between different isolates, rendering a *P* value (12), as well as for detecting the presence of a resistance gene, by studying the positions of the double-strand breaks of the individual plasmid barcodes within the consensus barcode (16).

### Whole-genome sequencing and bioinformatics.

Complete genomic DNA (chromosomal DNA and plasmids) was isolated using the Qiagen Genomic-tip 100 kit according to the manufacturer’s instructions. PacBio Sequencing was performed at the Science for Life Laboratory in Uppsala, Sweden, on a RSII system with one SMRT cell per genome. Illumina MiSeq sequencing was performed with two times 300-bp paired-end read lengths using Nextera XT DNA Library preparation kit (Illumina) according to the manufacturer´s instructions. The overall combined chromosomal coverage generated was >25-fold for all isolates.

*De novo* assembly was performed for each data set with the CLC Genomics workbench v.10 (CLC Bio, Aarhus, Denmark) including the CLC Microbial Genomics Module 3.0. Contig sequences from the PacBio sequencing were used as references for assembly of the Illumina reads, and the resulting contigs were circularized by detection of redundant end sequences and by mate-pair analysis. To verify that no plasmids were missed between isolates, *de novo* assembly of nonmatched reads from combined reference assemblies for each data set was performed, and remaining contigs were subjected to BLASTn searches and analyzed for gene content. The NCBI Prokaryotic Genome Annotation Pipeline was used for complete genome annotation. Detection of resistance genes, plasmid replicons, and virulence genes was done by submitting *de novo*-assembled contigs for each isolate to the online resources ResFinder (24), PlasmidFinder (25), and VirulenceFinder (26), respectively, at the Center for Genomic Epidemiology, DTU, Denmark.

### PCR.

PCR with primers flanking potential deletion breakpoints on the plasmids was performed. Amplification was done using purified plasmids as the template with the following procedure: initial denaturation at 98°C for 3 min, followed by 25 rounds of PCR with 1 round consisting of amplification by denaturation at 98°C for 30 s, annealing at 57°C for 30 s, and elongation at 72°C for 30 s. PCR products were analyzed on an agarose gel and visualized under UV light after ethidium bromide staining. PCR products were purified using ThermoFisher Scientific GeneJET Gel Extraction kit according to the manufacturer’s instructions and sent for Sanger sequencing at Eurofins Genomics (Germany) using the same primers as for the quantification of the deletion breakpoints. Primers are listed in Table S1 in the supplemental material.

10.1128/mBio.00347-19.6 TABLE S1 Primers for PCR analysis of the deletions in the 220-kb plasmids. Download Table S1, PDF file, 0.1 MB. Copyright © 2019 Bikkarolla et al. 2019 Bikkarolla et al. This content is distributed under the terms of the Creative Commons Attribution 4.0 International license.

### Data availability.

All sequences have been deposited at GenBank under BioProject PRJNA427077 with accession numbers CP025576 to CP025580.
