# Supplementary material for: Optical DNA Mapping Combined with Cas9-Targeted Resistance Gene Identification for Rapid Tracking of Resistance Plasmids in a Neonatal Intensive Care Unit Outbreak
Source: mBio. 2019 Jul 9;10(4):e00347-19. doi: 10.1128/mBio.00347-19 (PMC6747713; doi:10.1128/mBio.00347-19)
Supplement: FIG S1 [file mBio.00347-19-sf001.pdf]

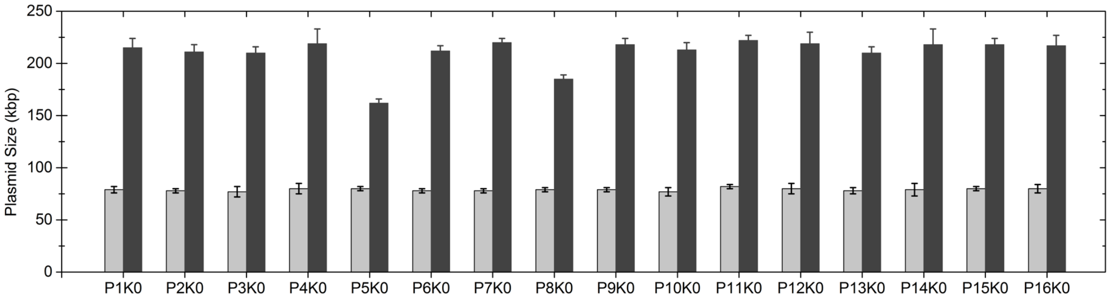

Figure S1. Sizes of plasmids in the ESBL-KP isolates at the start of the outbreak. The light grey bar represents the shorter shared plasmid and the dark grey bar represents larger shared plasmid.
