# Supplementary material for: Optical DNA Mapping Combined with Cas9-Targeted Resistance Gene Identification for Rapid Tracking of Resistance Plasmids in a Neonatal Intensive Care Unit Outbreak
Source: mBio. 2019 Jul 9;10(4):e00347-19. doi: 10.1128/mBio.00347-19 (PMC6747713; doi:10.1128/mBio.00347-19)
Supplement: TABLE S2 [file mBio.00347-19-st002.pdf]

Table S2. Statistical confirmation of Cas9 cut position for all EP-K ST101 80 kb plasmids.

Patient No	Cut Position (kbp)	No. of cuts at specific position	No. of cuts at random position	Maximum No. of cuts at specific position in a random case
P1K0	57.5	19	1	3.3 ± 0.7
P1K12	57.6	13	3	2.7 ± 0.65
P2K0	56.5	07	0	1.8 ± 0.56
P3K0	56	18	0	3.1 ± 0.7
P3K23	56	18	0	3.1 ± 0.68
P4K0	57	21	0	3.5 ± 0.7
P4K14	55.2	4	1	1.5 ± 0.55
P5K0	56.1	25	0	3.7 ± 0.7
P6K0	56.9	22	0	3.5 ± 0.72
P6K21	56.8	21	0	3.4 ± 0.71
P7K0	56.4	12	0	2.5 ± 0.6
P8K0	58.3	19	1	3.3 ± 0.68
P8K6	56.1	15	0	2.5 ± 0.62
P9K0	56.9	08	1	2.2 ± 0.54
P10K0	57	20	0	3.4 ± 0.72
P11K0	57.2	19	0	3.2 ± 0.68
P11K22	56.1	19	2	3.3 ± 0.69
P12K0	57.4	21	0	3.5 ± 0.73
P13K0	56.4	11	1	2.5 ± 0.6
P13K9	No Cas9 cut detected			
P14K0	56.6	9	1	2.3 ± 0.5
P15K0	56.5	20	0	3.3 ± 0.7
P16K0	56.7	08	4	2.6 ± 0.64
