# Supplementary material for: Optical DNA Mapping Combined with Cas9-Targeted Resistance Gene Identification for Rapid Tracking of Resistance Plasmids in a Neonatal Intensive Care Unit Outbreak
Source: mBio. 2019 Jul 9;10(4):e00347-19. doi: 10.1128/mBio.00347-19 (PMC6747713; doi:10.1128/mBio.00347-19)
Supplement: FIG S2 [file mBio.00347-19-sf002.pdf]

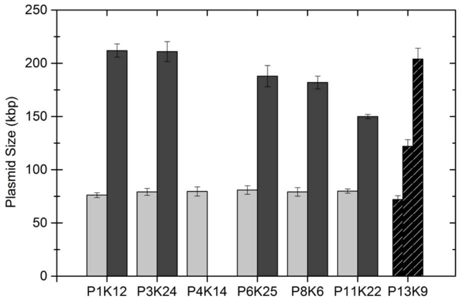

Figure S2. Sizes of plasmids in the ESBL-KP isolates in follow-up samples. The light grey bar represents the shorter shared plasmid and the dark grey bar represents larger shared plasmid. The dashed black bars represent the isolate that are unrelated to the other plasmids.
