# Supplementary material for: Optical DNA Mapping Combined with Cas9-Targeted Resistance Gene Identification for Rapid Tracking of Resistance Plasmids in a Neonatal Intensive Care Unit Outbreak
Source: mBio. 2019 Jul 9;10(4):e00347-19. doi: 10.1128/mBio.00347-19 (PMC6747713; doi:10.1128/mBio.00347-19)
Supplement: FIG S3 [file mBio.00347-19-sf003.pdf]

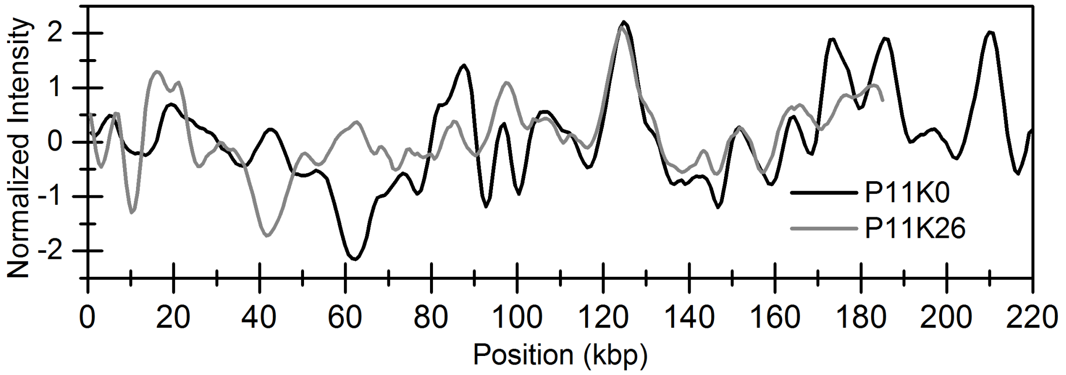

Figure S3. Structural variations in the large plasmid collected at 26 months in patient 11 compared to the same plasmid in the initial isolate.
