# Supplementary material for: Optical DNA Mapping Combined with Cas9-Targeted Resistance Gene Identification for Rapid Tracking of Resistance Plasmids in a Neonatal Intensive Care Unit Outbreak
Source: mBio. 2019 Jul 9;10(4):e00347-19. doi: 10.1128/mBio.00347-19 (PMC6747713; doi:10.1128/mBio.00347-19)
Supplement: TABLE S3 [file mBio.00347-19-st003.pdf]

Table S3. Descriptions of plasmid sequences from whole genome sequencing.

Isolate	Location	Size (bp)	MLST	Replicon[a]	Best match in GenBank (% identity/% query coverage)	Resistance gene/s[b]	Virulence genes/[c]
P1K0		215 872		$IncFIB_{K}$/IncFII	p69-1 (CP025457.1) (99/99)	*arsA-D, cop*-operon	*urtA-urtE, amiF*
		80 272		IncR/IncFIA	AR_0049 plasmid unitig_3 (CP018819.1) (81/99)	bla_{TEM-1A}, $bla_{CTX-M-15}$, *qnrB1*, *sul2*, *aac(3)-IId*, *aph(6)-Id*, *dfrA14*, *aph(3'')-Ib*	None
		4 572		ColRNAI	pJHCMW1 (AF479774.1) (58/92)	None	None
		4 163		ColRNAI	FDAARGOS_446 plasmid unnamed3 (CP023945.1) (100/100)	None	None
	Chromosome	5 277 535	ST101		ND	bla_{SHV-1}, *oqxA, oqxB*	ND
P3E6		129 195		IncFIB/ IncFII	pAA-ST131 plasmid ESBL20150001 (KY706108.1) (93/99)	None	*safC, afaB, afaD, aggR, agg5A*, ORF3, ORF4, *aap, aar, aatA*
		5 878		ColRNAI	pSL491_5 (CP001148.1) (100/97)	None	None
	Chromosome	5 425 761	ST38		ND	bla_{TEM-1B}, $bla_{CTX-M-15}$, *sul2*, *aac(3)-IIa*, *aph(6)-Id*, *dfrA1*, *aph(3'')-Ib*, *tet(D)*, *catA1*, *aadA1*	ND
