# Supplementary material for: Optical DNA Mapping Combined with Cas9-Targeted Resistance Gene Identification for Rapid Tracking of Resistance Plasmids in a Neonatal Intensive Care Unit Outbreak
Source: mBio. 2019 Jul 9;10(4):e00347-19. doi: 10.1128/mBio.00347-19 (PMC6747713; doi:10.1128/mBio.00347-19)
Supplement: FIG S4 [file mBio.00347-19-sf004.pdf]

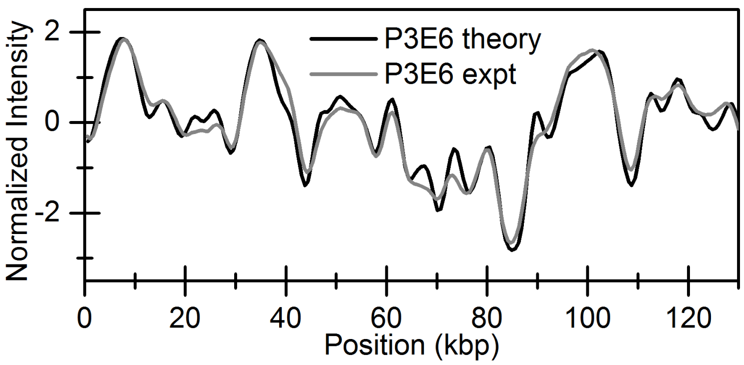

Figure S4. Comparison between experimental optical map (gray) and theoretical optical map obtained from PacBio sequencing (black) the 130 kbp plasmid in the *E. coli* isolate in patient 3.
