# Supplementary figures and images for: Optical DNA Mapping Combined with Cas9-Targeted Resistance Gene Identification for Rapid Tracking of Resistance Plasmids in a Neonatal Intensive Care Unit Outbreak
Source: mBio. 2019 Jul 9;10(4):e00347-19. doi: 10.1128/mBio.00347-19 (PMC6747713; doi:10.1128/mBio.00347-19)

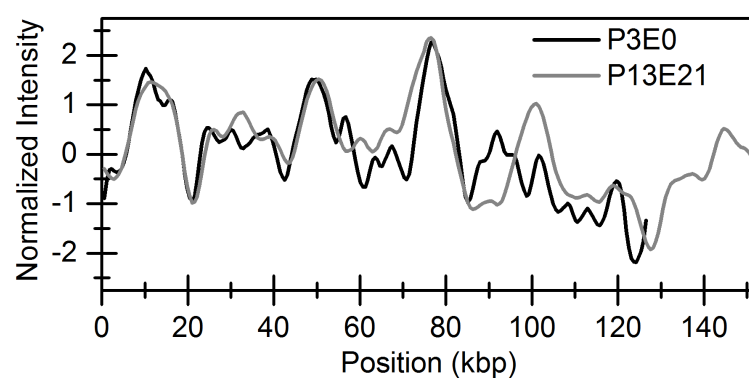

**Figure S5.** Similarity plot for 130 kbp plasmid in P3E0 and 152 kbp plasmid in P13E21.

Supplement: FIG S5 [file mBio.00347-19-sf005.pdf]
