# Supplementary material for: Optical DNA Mapping Combined with Cas9-Targeted Resistance Gene Identification for Rapid Tracking of Resistance Plasmids in a Neonatal Intensive Care Unit Outbreak
Source: mBio. 2019 Jul 9;10(4):e00347-19. doi: 10.1128/mBio.00347-19 (PMC6747713; doi:10.1128/mBio.00347-19)
Supplement: TABLE S1 [file mBio.00347-19-st001.pdf]

Table S1. Primers for PCR analysis of the deletions in the 220 kb plasmids

Strain	Primer	Sequence (5' → 3')
P3K0	P6F	ATCCATGGTGAGCATAGAAAC
	P14R	TAGTATCGCGAACCTCTT
P5K0	P7F-4	CATTTGTGCTGCACGAGTAC
	P8R	ATAGGGCGGAATACACCTTG
P6K21<br>P8K0<br>P8K6	P1F	CCAGCAAGCTAGGCAACATG
	P3R	GTCTGCTTATCGCTCACAAC
